# Role of prefrontal cortex in freezing of gait in Parkinson’s disease: mechanisms and neuroimaging evidence

**DOI:** 10.3389/fneur.2026.1713795

**Published:** 2026-02-17

**Authors:** Qiling Ji, Shanshan Mei, Lipeng Cai, Omar Elmadhoun, Yuchuan Ding, Xiaokun Geng

**Affiliations:** 1 Department of Neurology, Beijing Luhe Hospital, Capital Medical University, Beijing, China; 2 Department of Neurology, Xuanwu Hospital, Capital Medical University, Beijing, China; 3 China-America Institute of Neuroscience, Beijing Luhe Hospital, Capital Medical University, Beijing, China; 4 Division of Critical Care Medicine, Mayo Clinic, Rochester, MN, United States; 5 Department of Neurosurgery, Wayne State University School of Medicine, Detroit, MI, United States

**Keywords:** executive function, functional MRI, limbic system, neuroimaging, pathophysiological mechanisms

## Abstract

Freezing of gait (FOG) is a complex and highly disabling motor symptom that frequently affects patients with Parkinson’s disease (PD), especially in the middle and late stages. Although traditionally associated with motor deficits, accumulating evidence suggests that FOG is also strongly influenced by non-motor domains, including cognitive dysfunction and emotional disturbances. The prefrontal cortex (PFC), a key hub for executive functions, attention, and emotional regulation, has been increasingly implicated in the pathophysiology of FOG. Structural and functional abnormalities in the PFC, particularly in the dorsolateral prefrontal cortex (DLPFC) and medial prefrontal cortex (MPFC), have been reported in PD patients with FOG. This narrative review aims to summarize current evidence on the role of the prefrontal cortex in the development and modulation of freezing episodes, focusing on neuroimaging findings. Clarifying the contribution of frontal lobe dysfunction to FOG may inform therapeutic strategies targeting frontostriatal circuits to improve mobility and quality of life in PD patients. This is a narrative review and does not employ formal systematic review methodology.

## Introduction

Freezing of gait (FOG) is a paroxysmal motor symptom in Parkinson’s disease (PD) characterized by a sudden, brief inability or reduction in forward foot movement despite the intention to walk. Patients often describe it as feeling “glued to the floor.” FOG typically manifests during gait initiation, body turning, traversing confined narrow spaces, or under conditions of stress or dual-tasking. A 2010 consensus defined it as “a brief, episodic absence or marked reduction of forward progression of the feet despite the intention to walk” (1). Clinically, FOG can be classified into three motor phenotypes (2, 3): (1) trembling-in-place, marked by leg trembling (especially at the knees) during attempted gait initiation; (2) shuffling type, characterized by small, dragging steps with reduced stride length and (3) complete akinetic type, in which there is total immobility of the limbs. It may also be categorized by trigger: motor-induced (e.g., during transitions), cognitive-induced (e.g., dual-tasking), and emotional-induced (e.g., stress-related) (4). Based on the response of FOG to dopaminergic medication, FOG can be classified into off-state FOG, pseudo-ON-state FOG, dopa-unresponsive FOG, and rarer types including biphasic FOG and on-state FOG (3, 5–10). For off-state FOG and pseudo-ON-state FOG, the treatment strategy is to optimize dopaminergic medication. For on-state FOG, it is suggested to reduce dopaminergic medication. For biphasic FOG, management of dopaminergic treatment response fluctuations is particularly crucial. Dopa-unresponsive FOG is considered to be related to non-dopaminergic pathways, and non-pharmacological interventions should be adopted (6, 8, 11–13). These subtypes and their corresponding treatment approaches reflect the multifaceted nature of FOG and its complex pathophysiology involving motor, cognitive, and emotional domains. Accordingly, this review aims to synthesize current evidence on the role of the prefrontal cortex in freezing of gait, with particular emphasis on neuroimaging findings and mechanistic models.

## Search strategy/methods

This narrative review was informed by a targeted literature search of the PubMed and Web of Science databases. Articles published between 2000 and 2025 were identified using combinations of keywords including “Parkinson,” “Parkinson’s disease,” “freezing of gait”, and “gait.” Further relevant studies were identified via manual screening of reference lists from core articles. Specifically, the database search identified a total of 305 records (157 from PubMed and 148 from Web of Science). Titles and abstracts were screened for relevance to prefrontal cortex involvement, neuroimaging evidence, and mechanistic insights related to freezing of gait. Based on these predefined thematic criteria, 13 representative neuroimaging studies were selected for focused discussion and inclusion in Table 1. As this is a narrative review, study selection was guided by relevance and conceptual contribution rather than formal systematic inclusion/exclusion criteria.

**Table 1:** Neuroimaging studies in PD-FOG.

Author, year	Subjects	Dopaminergic state	Main findings in PD-FOG	Mechanistic model	Conclusions
Kostic, 2012 (74)	17 PD-FOG; 20 PD-nFOG; 34 HC	OFF	In PD-FOG, GM atrophy occurs in DLFPC, temporal, inferior parietal and occipital cortices, and FOG severity correlates with GM volume in the bilateral frontal and parietal cortices.	Using voxel-based morphometry to evaluate the extent and distribution of GM atrophy.	Frontal and parietal cortices structural damage induces executive dysfunction/perceptual deficits, which appears to be associated with FOG.
Shine, 2013 (78)	18 PD-FOG	OFF	The BOLD response in sensorimotor areas decreased, while that in frontoparietal cortical regions increased during freezing, and this response difference was negatively correlated with FOG severity.	VR gait paradigm-based fMRI for PD-FOG	The changes in blood oxygen level observed in the study suggest that sensorimotor regions, frontoparietal cortical regions, caudate nucleus head, thalamus, globus pallidus and the MLR are related to the onset of FOG.
Fling, 2013 (73)	14 PD-FOG; 12 PD-nFOG; 15 HC	OFF	The connectivity between the right PPN and cerebellum, thalamus, and frontal cortex is decreased in PD-FOG.	DTI was used to quantify PPN structural connectivity.	FOG is closely associated with structural abnormalities of the right hemispheric PFC, which mediates executive inhibitory functions.
Shine, 2013 (76)	10 PD-FOG; 10 PD-nFOG	OFF and ON	Functional decoupling between bilateral basal ganglia network and cognitive control network in PD-FOG patients.	fMRI data acquired/analyzed during VR gait tasks (dopaminergic medication: ON/OFF states) in patients.	Impaired communication between complementary yet competitive neural networks contribute to FOG occurrence in PD-FOG patients.
Canu,2015 (77)	22 PD-FOG; 35 HC	OFF and ON	WM impairment in the PFC; reduced functional connectivity of frontoparietal regions within the DMN in PD-FOG.	T1-weighted, DT MRI and RS fMRI	Poor structural-functional integration between motor and cognitive systems may be the cause of FOG.
Pietracupa, 2018 (75)	21 PD-FOG; 16 PD-nFOG; 19 HC	OFF	In PD-FOG, the CTh in right DLPFC lower than that in HS, and the WM in the radiation area of frontal lobe changes significantly.	MRI was performed, and gray matter and white matter were evaluated.	High-level gait control cortical area damage and WM disruption (motor/cognitive/limbic structures) correlates with PD-FOG anatomical basis.
Gilat, 2018 (80)	19 PD-FOG; 21 PD-nFOG	OFF	In PD-FOG, increased FPN-left amygdala anti-coupling and decreased FPN-right putamen anti-coupling; functional interactions correlate with FOG severity and fear of falling.	Rs-fMRI applied to study inter-brain network functional connectivity differences.	The association between anxiety and FOG in PD may be mediated by dysfunction of the fronto-striato-limbic system pathway.
Li, 2020 (41)	25 PD-FOG; 30 PD-nFOG; 26 Hc	ON	Enhanced FC in advanced cognitive-attention networks in PD-FOG patients.	ICA assesses brain interconnection differences following rs-fMRI.	PD-FOG mechanisms correlate with advanced cognitive and attention network alterations.
Li, 2021 (38)	37 PD-FOG; 38 PD-nFOG	OFF	PD-FOG showed reduced node centrality in the FPN and cerebellum, but elevated node centrality and FC in SMN, FPN, VN, subcortical and limbic regions.	Rs-fMRI builds whole-brain functional networks and assesses topological characteristics via graph theory.	PD-FOG arises from multi-network dysfunction.
Taylor, 2022 (83)	29 PD-FOG	OFF	Under threat conditions, crosstalk within and between the motion, limbic, and cognitive networks is augmented, causing increased FOG.	Elicit anxiety via virtual plank navigation with simultaneous task-based fMRI in PD-FOG.	Anxiety may drive a specific subtype of FOG in Parkinson’s disease.
Quek, 2024 (79)	23 PD-FOG	OFF and ON	Anxiety correlates with left amygdala-PFC and left putamen-PPC rsFC.	Rs-fMRI assessed rsFC linked to FOG in PD under unmedicated and medicated states.	Dopaminergic medications may mediate the partial modulation of the frontoparietal-limbic-striatal circuitry in PD-FOG.
Cockx, 2024 (39)	25 PD-FOG; 24 HC	OFF	PD-FOG exhibit lower PFC activity during freezing vs. spontaneous stops; supplementary motor area SMA-PFC activity related to motor cessation is unbalanced in freezing states.	FNIRS assesses FOG-related cortical haemodynamics (freely moving)	Paroxysmal imbalance between SMA and PFC leads to FOG.
Taniguchi, 2024 (40)	41 PD-FOG; 41 PD-nFOG	ON	PD-FOG show abnormally enhanced rsFC between bilateral DLPFC/MLR; left DLPFC independent self-inhibition connectivity negatively with FOG severity.	Rs-fMRI spectral DCM	PD-FOG reduction may be achieved by targeting left DLPFC-MLR effective connectivity.

PD-FOG, Parkinson’s disease patients with freezing of gait; PD-nFOG, Parkinson’s disease patients without freezing of gait; HC, healthy control people; HS, healthy subjects; ON, on medication; OFF, off medication; BOLD, blood oxygenated level-dependent signal; WM, white matter; GM, gray matter; CTh, cortical thickness; PFC, prefrontal cortex; DLPFC, dorsolateral prefrontal cortex; MLR, mesencephalic locomotor region; PPC, posterior parietal cortex; PPN, pedunculopontine nucleus; VR, virtual reality; DMN, default mode network; FC, functional connectivity; ICA, independent component analysis; FPN, frontoparietal network; SMN, sensorimotor network; SMA, supplementary motor area; DCM, dynamic causal modeling; DT, diffusion tensor; DTI, diffusion tensor imaging; VN, visual network; fMRI, functional MRI; rs-fMRI, resting-state fMRI; rsFC, resting state functional connectivity; fNIRS, functional near-infrared spectroscopy; RSNs, resting-state networks; ICA, independent component analysis.

## Epidemiology of freezing of gait (FOG)

The prevalence of FOG in PD patients varies widely, from 7 to 60%, depending on the disease stage, diagnostic criteria, and assessment tools (14, 15), with incidence increasing over time. FOG is one of the most disabling motor symptoms in PD, particularly in the middle to advanced stages (16). Giladi et al. (17) found that over 50% of patients with more than 5 years of PD experience FOG. It is particularly common among those with the postural instability and gait difficulty subtype, compared to those with tremor-dominant forms. Studies have shown that FOG in PD patients correlates with longer disease duration, higher apathy scores, higher UPDRS II and III scores, higher daily levodopa equivalent dose, and more frequent exposure to antimuscarinics (18). It contributes significantly to falls, which affect up to 70% of PD patients, often resulting in injury, loss of confidence, and reduced independence (19). FOG is also associated with lower quality of life, particularly in mobility and daily living domains.

A recent systematic review by Bansal et al. (20) summarizes current techniques for the detection and management of FOG, highlighting the limitations of traditional clinical scales and the increasing role of wearable sensor-based gait analysis. These emerging technologies provide objective, high-resolution assessment of FOG and have important implications for both research and clinical practice. Evidence suggests that beyond basal ganglia dysfunction, FOG is also closely related to impairments in cognitive, emotional, and executive function domains mediated by the prefrontal cortex (PFC) (21–27). Neuroimaging and behavioral studies implicate PFC abnormalities, particularly in contexts involving cognitive load, anxiety, or spatial constraints (28–31).

## Pathophysiological mechanisms of freezing of gait (FOG)

Understanding the underlying pathophysiology of FOG remains one of the most challenging aspects of PD research. Normal gait depends on the coordinated interaction of multiple neural systems operating across different hierarchical levels (32). The peripheral nervous system, central pattern generators (CPGs), and musculoskeletal system, support low-level automatic locomotor functions. In contrast, supraspinal “locomotor regions of the brain,” including the subthalamic locomotor region (SLR), mesencephalic locomotor region (MLR), and the cerebellar locomotor region (CLR), are responsible for activating and regulating CPG output during gait initiation, termination, and rhythmic stepping. At the higher level, the cerebral cortex and basal ganglia (BG) exert both direct and indirect control over these locomotor regions, enabling flexible, goal-directed, and context-dependent gait regulation (33). Disruption at any connection within this distributed network can impair locomotor automaticity and precipitate freezing episodes (33–42). Lesions associated with freezing of gait have been reported in diverse brain regions, including parasagittal frontal areas, cerebellum, postcentral gyrus, brainstem, midbrain tegmentum, and basal ganglia (34). The anatomical heterogeneity of these lesions supports the concept that freezing of gait arises from dysfunction within a widely distributed and interconnected locomotor network, rather than from a single focal structure. Five major pathophysiological models have been proposed to account for the occurrence of FOG. Neuroimaging studies provide support for three of these models: (i) the “interference model,” which proposes that excessive compensatory cortical recruitment during dual-task walking overwhelms limited executive resources (43, 44); (ii) the “perceptual dysfunction” model, which attributes FOG to impaired dorsal visuomotor processing that interferes with online motor planning and spatial navigation (45); and (iii) the “executive dysfunction” model, which links FOG to impaired connectivity between the frontal lobe and basal ganglia. In this model, abnormal activation of executive regions may represent compensatory responses under conditions of increased attentional demand. These mechanisms are not mutually exclusive and likely interact. In contrast, the decoupling and abnormal gait pattern generation models have not been consistently supported by neuroimaging evidence (46, 47).

Neuroimaging studies support a multifaceted pathophysiology involving cognitive, affective/limbic, and motor control domains (4, 48). These dysfunctions may exceed the striatum’s processing capacity in PD, leading to hyperactivity of the globus pallidus interna (GPi) and excessive inhibition of the brainstem locomotor centers—including the pedunculopontine nucleus (PPN), mesencephalic locomotor region (MLR), and cerebellar locomotor region (CLR), which together give rise to the occurrence of FOG episodes (43).

Clinically, FOG manifests as impaired gait rhythm, initiation hesitation, reduced step length and speed, and postural instability (1). Triggers include distractions, dual-tasking, or the need to rapidly switch motor program (49). Compared with PD patients without FOG, those with FOG perform more poorly on cognitive tasks, especially those involving executive function, attention, and visuospatial abilities (50, 51). Emotional triggers are also common, FOG frequently arises under stress, in narrow spaces, or when walking in darkness. Anxiety and depression are prevalent in PD and have been independently linked to increased FOG severity and frequency (52–56).

## Movement, cognition, limbic system in prefrontal cortex

**Figure 1:**
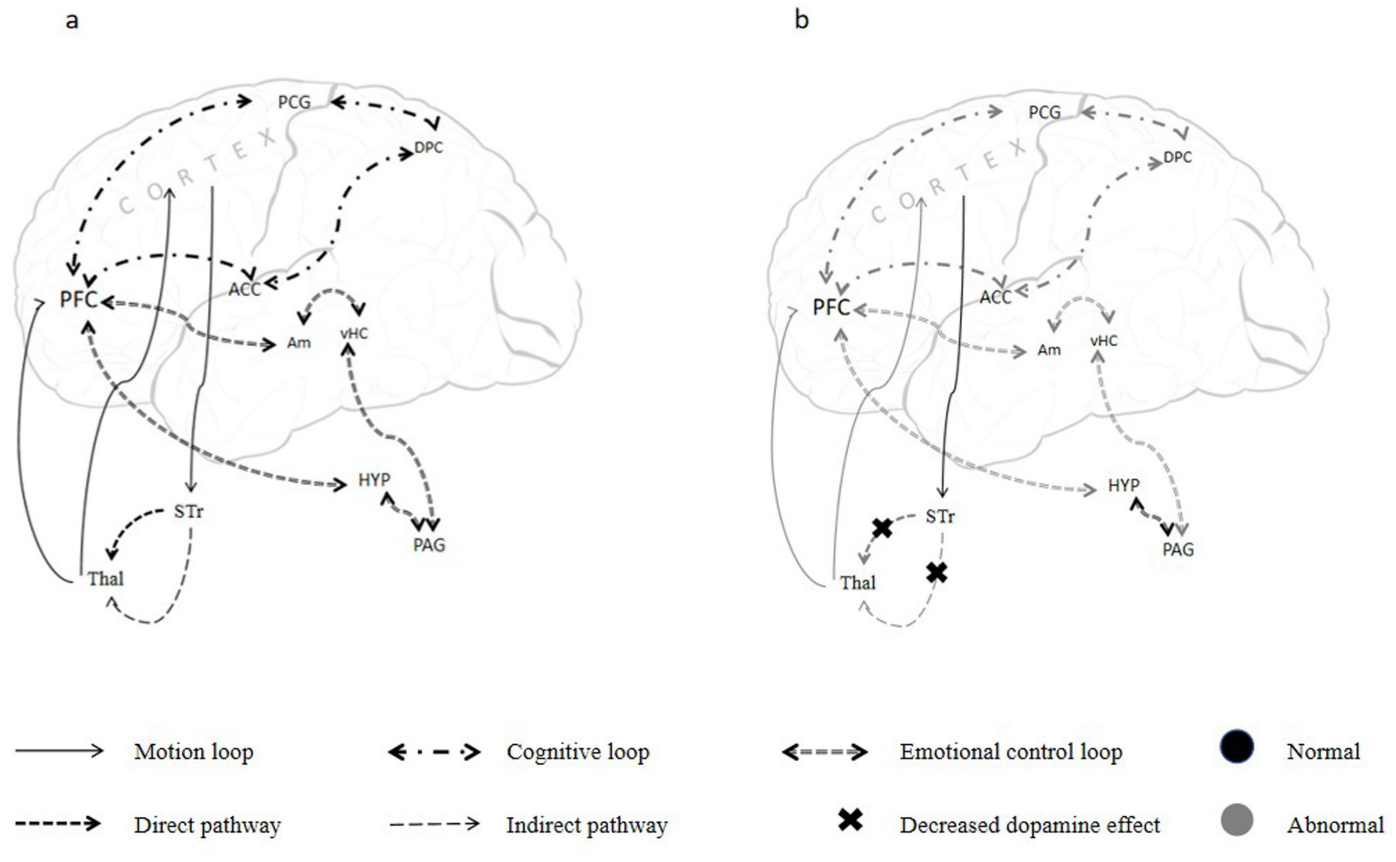
Neural pathways for **(a)** Normal Gait and **(b)** Freezing of Gait. Excitatory fibers from extensive regions of the cerebral cortex project to the basal ganglia circuitry, which subsequently projects to the premotor cortex and prefrontal cortex. The prefrontal cortex and limbic system circuits collectively form the cognitive control circuitry, jointly supporting higher cognitive functions such as working memory and selective attention. The emotional control circuitry is similarly composed of complex neural pathways. Disorders in the striato-thalamic-prefrontal, cortico-limbic, and mediotemporal-limbic networks lead to the emergence of depressive states. Motor control impairments, executive and cognitive dysfunctions, as well as abnormal emotional states, contribute to the development of FOG. PFC, prefrontal cortex; Thal, thalamus; DPC, dorsal parietal cortex; PCG, precentral gyrus; ACC, anterior cingulate cortex; PAG, periaqueductal grey; HYP, hypothalamus; vHC, ventral hippocampus; Am, amygdala; STr, striatum.

The frontal lobe is a critical region of the human brain and is broadly divided into three parts: motor cortex, pre-motor cortex and prefrontal region. The prefrontal cortex (PFC) is composed of the dorsal, ventrolateral, preorbital, and ventromedial areas of the frontal cortex (57). PFC is responsible for complex cognitive behavior, decision making, and social behavior, and operates through intricate subcortical circuits. Currently, five frontal–subcortical circuits have been well described, all involving the prefrontal cortex and basal ganglia circuits. Among these, the motor circuit governs motor functions; the oculomotor nerve circuit regulates eye movements; and the orbitofrontal subcortical circuits, dorsolateral prefrontal-subcortical circuit, and medial prefrontal-subcortical circuit are involved in cognitive, emotional, and motivational processes (58) (Figure 1).

### Motor regulation in prefrontal cortex

The PFC also plays a role in motor regulation. The pre-motor cortex (PMC) and primary motor cortex (M1) are connected to several prefrontal regions, enabling cognitive control of motor behavior, emotional influences on action execution, and integration with spinal motor output. Brain imaging studies have shown that reduced volume in prefrontal regions is associated with impaired gait performance. In PD patients with exercise inability, decreased activation has been observed in several prefrontal areas, including the dorsolateral prefrontal cortex (DLPFC) and inferior frontal gyrus (IFG) (59). Using [18F]-2-fluoro-2-deoxyglucose positron emission tomography (FDG-PET) and scaled subprofile model/principal components analysis (SSM/PCA), researchers have identified that gait-related networks involving pace and average step size are associated with region-specific increases and decreases in metabolic activity across the frontal cortex including the DLPFC, orbital frontal lobe, and auxiliary motor area (60).

### Executive function in prefrontal cortex

The PFC is central to executive function. Within this domain, the DLPFC is a key region involved in working memory, inhibition, fluency and executive function (61, 62). Cognitive impairment in PD typically affects executive function, attention control, decision making, language fluency and response inhibition. Multiple functional imaging studies have confirmed prefrontal dysfunction in PD. Functional magnetic resonance imaging (fMRI) reveals significantly reduced PFC activity during task performance, with insufficient activation of the frontoparietal network. These changes are more pronounced in PD patients with cognitive impairment (63). Structural imaging studies combining postmortem *in situ* MRI and histopathology have shown altered DLPFC microarchitecture in patients with PD, Parkinson’s disease dementia (PDD), and dementia with Lewy bodies (DLB) (64). In addition, resting-state functional connectivity (FC) analyses have identified reduced FC within the DLPFC (65) and between the DLPFC and cerebellar regions. Some studies have also shown improved FC between the right IFG and left DLPFC, suggesting potential compensatory mechanisms (66).

### Emotional regulation in prefrontal cortex

The PFC also plays a critical role in emotional regulation (67–70). A systematic review of neuroimaging studies investigating apathy, depression, and anxiety in PD identified the frontotemporal circuitry as a common pathway for these symptoms (71). Several imaging studies have linked depression in PD to dysfunction in the DLPFC. One study using 53-channel functional near-infrared spectroscopy (fNIRS) to assess brain network characteristics in PD patients with depressive symptoms (PD-D) found significantly reduced interhemispheric FC, particularly between the left DLPFC and the left frontal polar area (72). Other research has suggested not only underactivation of the left DLPFC, but also an imbalance in frontal activity. According to the “frontal asymmetry” hypothesis, depression is associated with a relative imbalance in activity between the left and right frontal regions (71).

## MR-confirmed changes of the prefrontal cortex in Parkinson’s patients with freezing of gait

Structural and functional abnormalities in the PFC have been consistently reported in PD patients experiencing FOG, as demonstrated by various neuroimaging studies (Table 1). PD patients exhibit FOG during executive dysfunction, and diffusion tensor imaging (DTI) reveals reduced connectivity between the pedunculopontine nucleus (PPN) and PFC (73). MRI studies show PD-FOG patients exhibit significant GM atrophy in multiple cortical regions (including dorsolateral prefrontal cortex) and notable white matter (WM) changes, compared with healthy controls (74, 75). The severity of FOG was found to correlate with GM volume in both the bilateral frontal and parietal cortices (74). A randomized controlled trial using functional MRI (fMRI) revealed functional disconnection between the basal ganglia network and cognitive control network in both hemispheres during task performance—an alteration associated with episodes of paroxysmal motor arrest (76). Resting-state (RS) fMRI studies have likewise demonstrated reduced functional connectivity within fronto-parietal regions of the default mode network and the occipital cortex within the visual association network, abnormally enhanced functional connectivity between the bilateral DLPFC and the bilateral medial regions (MLR), as well as increased functional connectivity in higher-order cognitive and attention-related networks in PD-FOG patients (40, 41, 77). Task-based MRI studies using a virtual reality paradigm have suggested that frontal-striatal network overload disrupts gait during cognitive tasks. Complementary findings from functional near-infrared spectroscopy (fNIRS) studies have shown heightened PFC activity in PD patients with FOG during both single- and dual-task walking and during single-task steering, with increased activation correlating with worse FOG metrics (33, 42, 78). But a study have reported an opposite conclusion, suggested that freezing arises from a paroxysmal imbalance between the SMA and the PFC (39).

Additionally, RS-fMRI research has shown that simulated “walking” under high-threat conditions is correlated with heightened functional connectivity—or “crosstalk”— between amygdala and fronto-parietal networks (FPN) in FOG-positive versus FOG-negative PD patients (79). In the same study, connectivity between the putamen, amygdala, and FPN markedly correlated with both fear of falling and FOG severity. These findings provide some of the first direct evidence linking dysfunctional fronto-striatal-limbic interactions to the association between anxiety and FOG in PD (80). DLPFC, in particular, is a key component of the prestriatal pathway linking the frontal cortex to the striatum (81, 82). A study using an fMRI-based anxiety-inducing VR gait paradigm further explored the role of anxiety in the development of FOG. It revealed increased intra- and inter-network functional activity between the PFC and motor, limbic, and cognitive networks, suggesting that anxiety-related sympathetic arousal might facilitate hyperconnectivity among distributed cortical networks, ultimately contributing to paroxysmal FOG episodes (83).

## Novelty and significance

This review provides a novel and integrative perspective on FOG in PD by positioning the PFC as a central hub linking motor, cognitive, and limbic dysfunction within a distributed locomotor network. Unlike prior reviews that primarily catalog neuroimaging findings, the present work systematically maps imaging evidence onto established mechanistic models of FOG, including interference, executive dysfunction, perceptual dysfunction, and limbic network involvement. By synthesizing recent advances from task-based and resting-state fMRI, structural imaging, and functional near-infrared spectroscopy, we advance a network-level framework that explains how cognitive load, emotional stress, and impaired top–down control converge to precipitate freezing episodes. Importantly, this review highlights emerging evidence implicating prefrontal–striatal–limbic interactions, particularly under conditions of anxiety and dual-tasking, thereby extending traditional basal ganglia–centric views of FOG. By identifying current methodological limitations and unresolved mechanistic questions, this work also delineates clear directions for future research and supports the development of targeted, circuit-based therapeutic strategies aimed at improving gait and quality of life in patients with Parkinson’s disease.

## Discussion

Despite these compelling observations, most existing studies provide only indirect, primarily correlational, evidence regarding the PFC’s role in FOG. It should be emphasized that most existing neuroimaging studies of FOG are cross-sectional and correlational in nature, which limits causal interpretation. It remains unclear whether the observed PFC abnormalities directly cause FOG initiation, reflect compensatory mechanisms for abnormal motor and non-motor symptoms lead to PFC functional overload, or arise secondary to repeated FOG. In addition, PFC hyper- or hypo-activation may represent adaptive or maladaptive network reorganization rather than primary pathology. Alternatively, it is possible that multiple mechanisms coexist and interact dynamically over the course of disease progression. Longitudinal, interventional, and multimodal studies will be essential to disentangle causality and clarify the precise role of prefrontal circuits in FOG pathophysiology. Further investigation is needed to clarify the specific contribution of the PFC to FOG pathophysiology. Given the growing body of behavioral and neuroimaging evidence implicating prefrontal dysfunction, such research may guide the development of novel therapeutic strategies to mitigate FOG in patients with Parkinson’s disease.
